# Modulation of Central Nociceptive Transmission by Manual Pressure Techniques in Patients with Migraine: An Observational Study

**DOI:** 10.3390/jcm11216273

**Published:** 2022-10-25

**Authors:** Willem De Hertogh, Andreas Amons, Lise Van daele, Ellen Vanbaelen, René Castien

**Affiliations:** 1 Department of Rehabilitation Sciences and Physiotherapy, Faculty of Medicine and Health Sciences, University of Antwerp, Universiteitsplein 1, 2610 Wilrijk, Belgium; 2 Department of General Practice, Amsterdam Public Health Research Institute, Vrije Universiteit Amsterdam, De Boelelaan 1117, 1118, 1081 HV Amsterdam, The Netherlands; 3 Health Care Center, Waddenweg 1, 2134 XL Hoofddorp, The Netherlands; 4 Faculty of Behavioural and Movement Sciences, Vrije Universiteit Amsterdam, Amsterdam Movement Sciences, De Boelelaan 1105, 1081 HV Amsterdam, The Netherlands

**Keywords:** migraine, neck pain, referred pain, neck muscles, upper cervical spine, physical examination

## Abstract

Background: Manual pressure in the upper cervical spine is used to provoke and reduce the familiar migraine headache. Information is scarce on the segmental levels, myofascial structure provocation, and reduction occurrences. The required dosage (amount of pressure, number of repetitions, and duration) has not been objectified yet. Methods: Prospective observational study. Thirty patients with migraine were examined interictally. Manual pressure was applied at four sites: the posterior arch of C1, the articular pillar of C2, the rectus capitis posterior major muscle, and the obliquus capitis inferior muscle, bilaterally. On sites where the familiar headache was provoked, the pressure was sustained to induce pain reduction (three repetitions). Provocation of familiar headache (yes/no), headache intensity (numerical pain rating scale), time to obtain a reduction of the headache (seconds), and applied pressure (g/cm^2^) were recorded. Results: Provocation of the familiar headache occurred at the posterior arches C1 in 92%, and at one of the articular pillars of C2 in 65.3% of cases. At one of the rectus capitis major muscles, the familiar headache was provoked in 84.6% of cases; at one of the oblique capitis inferior muscles, the familiar headache was provoked in 76.9% of cases. The applied mean pressure ranged from 0.82 to 1.2 kg/cm^2^. Maintaining the pressure reduced headache pain intensity significantly between the start and end of each of the three consecutive trials (*p* < 0.04). This reduction occurred faster in the third application than in the first application (*p* = 0.03). Conclusion: Manual pressure at upper cervical segments provokes familiar referred migraine headaches, with low manual pressure. Maintaining the pressure reduces the referred head pain significantly, indicating modulation of central nociceptive transmission.

## 1. Introduction

A migraine is a common primary headache type with an estimated global prevalence of 15.3% [1]. As a recurrent and often life-long headache disorder, migraine has high personal and socioeconomic impacts [2,3]. Besides pharmacological interventions, non-pharmacological interventions, such as relaxation training, cognitive behavioral therapy, and non-invasive neurostimulation, are efficient approaches to treat migraine [4,5]. Most physical treatments are targeted at the neck [6]. This is not surprising, as people with migraine frequently report neck pain in the pre-, post, and ictal phases of the migraine attack [7,8,9]. This co-occurrence of headache and neck pain could be explained by common nociceptive innervation of the head and neck at the trigeminal–cervical complex [10,11,12]. This merge of nociceptive innervation at the trigeminal–cervical complex (irrespective of whether neck pain is a symptom or a contributing factor of the migraine attack), provides a rationale for referred pain from the neck to the head as well as for interventions targeted at the cervical spine [13,14].

Referred pain to the head can be provoked by pressure or stretches on cervical myofascial structures, such as muscles, ligaments, and joints. Provocation of headaches by techniques using a combination of movement and manual pressure on upper cervical myofascial structures may distinguish patients with migraine from healthy controls [14,15,16]. Repetition and sustained pressure during the application of these specific techniques at the upper cervical spine may also reduce referred pain to the head and appear to be prognostic factors for the success of the physiotherapy treatment of patients with migraine [16]. The intensity of the referred headache seems to be affected by the repetition of these techniques and induces a change in the brainstem by altering the nociceptive blink reflex [17].

Although provocation and reduction of referred head pain can be used to assess and treat patients with headaches, information about the applied pressure during these tests and the relevance of these specific tests is missing.

Our study explores if familiar headaches can be provoked and reduced by manual pressure at the upper cervical spine in patients with migraine. The pressure to obtain this provocation as well as the reduction of referred pain have not been objectified as yet. Therefore, we measure the applied pressure and the immediate effects on referred pain to the head during the application of these techniques in patients with migraine. This information is mandatory to gain further insight into the underlying pathophysiological mechanisms and to verify if a change in the applied pressure does not cause the observed decrease. 

The objectives of this study were to determine whether manual pressure can provoke referred pain to the head in migraine, verify if sustained manual pressure can reduce referred pain to the head, and constantly monitor the amount of pressure.

## 2. Materials and Methods

### 2.1. Design

A prospective observational study was conducted and reported according to the STROBE criteria for observational studies (https://www.strobe-statement.org, accessed on 1 January 2022). 

### 2.2. Patients

Patients with migraine were recruited from primary healthcare centers that specifically focused on headaches and neck pain. All patients were initially screened for eligibility by a telephone interview using a structured questionnaire. Patients were diagnosed according to the criteria of the International Classification of Headache Disorders (ICHD III) by a general practitioner or neurologist [18].

Patients were excluded in case of other headaches, medication overuse, rheumatoid disorder, chronic diseases (e.g., fibromyalgia), a recent history of neck/head trauma (e.g., whiplash), anesthetic block in the past month, pregnancy, and symptoms of concomitant illness. Furthermore, migraine needed to be accompanied by neck pain; a good understanding of the Dutch language was required as all information, instructions, and questionnaires were in Dutch. Each patient was asked to keep a headache diary for four weeks to confirm the diagnosis and frequency of the migraine. Measurements were planned interictally, and patients were not allowed to take analgesics or muscle relaxants twenty-four hours before the examination.

### 2.3. Measurements

Measurements consisted of the completion of the headache diary (headache frequency and headache intensity), two questionnaires (headache impact tests, HIT-6, and central sensitization inventory), the assessment of pressure pain thresholds (PPTs), and measurement of pressure during MPT.

#### 2.3.1. Headache Characteristics

The headache diary was used to calculate the headache frequency (days/month) and mean headache intensity on a numerical pain rating scale (NPRS). The NPRS is a scale with 11-points, with 0 indicating no pain and 10 indicating intense pain (as intense as one could imagine) [19,20]; it was used to rate the intensity of the headache and neck pain. The mean headache intensity was calculated by dividing the sum of all NPRS scores on headache days and the mean of all noted headache days.

#### 2.3.2. Questionnaires

The headache impact test 6 (HIT-6) measures the adverse impacts of headaches on social functioning, role functioning, vitality, cognitive functioning, and psychological distress; it also measures the severity of the headache pain. This questionnaire consists of six items that need to be answered on a five-point scale ranging from never to always. The total score varies between 36 and 78, with a higher score meaning a more significant impact. HIT-6 scores are divided into four severity levels; little or no impact (≤49), some impact (50–55), substantial impact (56–59), and severe impact (≥60). The HIT-6 is equally reliable (and valid) in patients with headaches and chronic migraine [21,22].

The central sensitization inventory (CSI) is also a self-reported scale designed to highlight the possible presence of a central sensitization syndrome. The first part, Part A, assesses 25 health-related symptoms common to central sensitization syndromes. Total scores range from 0 to 100, where a cut-off score of 40 provides a clinically relevant guide to assume the possible presence of a central sensitization syndrome [23]. Part B collects the presence of previous diagnoses of seven separate central sensitization syndromes, including migraine. The Dutch version used in this study had good internal consistency for the total score in 3 out of 4 domains, good discriminative power, and excellent test-retest reliability [24]. 

#### 2.3.3. Pressure Pain Thresholds

The PPTs were assessed with the Somedic algometer with a 1 cm^2^ probe and are expressed in kPa/cm^2^. This algometer has excellent construct validity and high intra-rater reliability in people with migraine [25]. This was conducted by examiner 1 (E.V.), who had 10 hours of training for these measurements. In total, three measurements were performed on the midpoint of the upper trapezius, thenar, and anterior tibial anterior muscle, bilaterally. The pressure gradually increased (50 kPa/s) until the feeling of only pressure changed into the feeling of pressure and pain. 

The participant had to push the button of a hand-held switch. Once the participant pushed the button, the recorded value on the display of the algometer was noted [25].

#### 2.3.4. Manual Pressure Techniques

The MPTs were performed by two musculoskeletal physiotherapists (W.D.H. and R.C.); each has over 20 years of experience in assessing and treating patients with cervical spine disorders. Four techniques were performed bilaterally. 

The first two techniques have been described by Watson et al. [14]. The patient lies in a supine position, and pressure with the thumb is applied at the posterior arch of C1, with the participant’s head in approximately 20 degrees of contralateral rotation. By adding a slight rotation of the head toward the thumb, stress is applied to the joint of C0-1. In preparation for the second technique, pressure with the thumb was directed to the articular pillar of C2 with the participant’s head in approximately 30 degrees of contralateral rotation to passively stress the joint of C2-3. 

**Figure 1:**
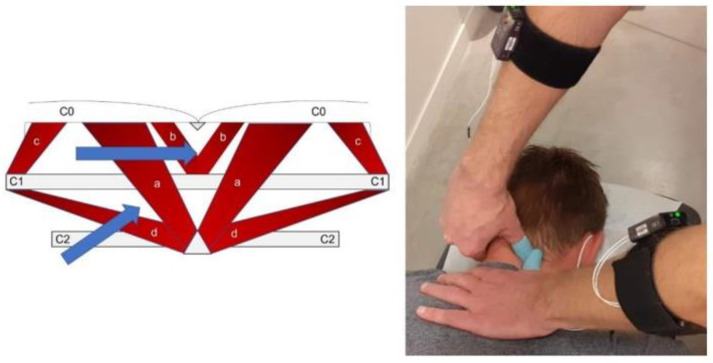
Schematic presentation of the suboccipital muscles (**left**) and demonstration of the stretch of the rectus capitis posterior (**right**). Blue arrows represent directions of the applied stretch on the rectus capitis posterior (a) and the obliquus capitis inferior (d). Moreover, represented: rectus capitis posterior minor (b) and obliquus capitis superior (c).

The third and fourth techniques were performed with the patient in a prone position and the cervical spine in a neutral position. Pressure with the thumb was applied deep toward the occiput to stretch the rectus capitis major muscle (the third technique). At a lower level, pressure with the thumb was applied toward the spinous process of C2 and attempted to stretch the obliquus capitis muscles (the fourth technique). Schematic representations of techniques 3 and 4 are presented in Figure 1.

The outcome of these MPTs was defined as positive (yes/no) if provocation of the familiar headache occurred within 5 seconds. In all positive cases, the intensity of the provoked pain was registered via an NPRS.

#### 2.3.5. Registration of the Applied Thumb Pressure

**Figure 2:**
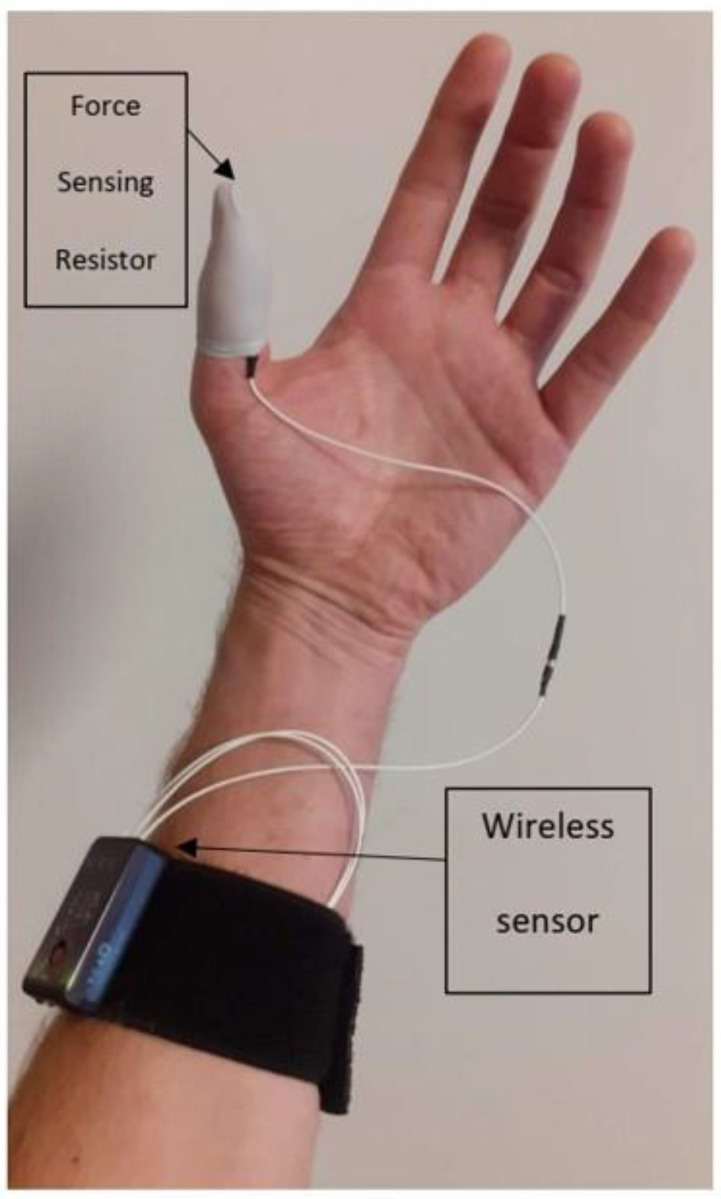
Experimental setup with the location of the force sensing resistor (FSR) sensors on the top of the thumb.

During the MPT, the thumb pressure of the assessor was measured constantly using force-sensing resistor sensors (1.23 cm^2^) that were placed on the tip of the thumb (Figure 2) and registered by CAPTIV software (CAPTIV-L7000, www.teaergo.com, accessed on 1 January 2022). These sensors measure the applied pressure in kg/cm^2^. During the examination, a third assessor was in control of the software so that the examiner who performed the MPT was blinded to the outcome of the applied pressure. The third assessor also recorded the patient’s reported outcome of the MPT.

#### 2.3.6. Procedures

The NPRS scores for headache and neck pain were obtained before the measurements. When the NPRS scores were ≤3/10, it was considered that the pain did not influence the tests. PPTs were measured.

The MPT measurements were performed in two steps. In the first step, we verified if (and at which site) the familiar headache could be provoked. The outcomes of the MPTs being provocation of headache (yes/no) and corresponding headache intensity (NPRS score) were recorded. 

In the second step, the MPTs were performed and maintained for both the provocation and reduction of the referred pain to the head. The pressure was maintained until the NPRS score of the headache was reduced to <2/10 or after a maximum of 120 seconds. During the application of the MPTs, the NPRS scores were noted every 15 seconds and the applied pressure was registered constantly via the FSR sensors.

Sufficient time was left between the two steps so there was no residual pain.

### 2.4. Sample Size Determination

A previous study included 15 participants [17]. However, concrete means and standard deviations were not reported. Only F-test and *p*-values were given, and a graphical representation of the numbers. Consequently, no numerical data were available for a sample size calculation. Therefore, we doubled the sample size of the previous study and included 30 participants.

### 2.5. Data Analysis

Data were analyzed with the SPSS version 27 software (IBM Corp. Released 2020. IBM SPSS Statistics for Windows, Version 27.0. Armonk, NY, USA: IBM Corp).

Descriptive data and headache characteristics were collected from all patients. The descriptive analysis was also performed for the outcomes of the MPT. 

The normality of quantitative data was assessed by means of the Kolmogorov–Smirnov test. Parametric tests were used for quantitative data with a normal distribution. Conversely, non-parametric tests were used for qualitative data and quantitative data without normal distribution.

To study the reduction of the provoked headache, comparisons of NPRS scores before and after each trial were made via (paired samples T-test or) and across all three trials via a Friedman test. Changes in the time needed to obtain the reduction were analyzed via a Friedman test and consequent Mann–Whitney U tests. 

A 2-tailed *p*-value < 0.05 was chosen as the level of significance.

## 3. Results

### 3.1. Demographic Data and Headache Characteristics

A total of 30 patients with migraine, 3 men and 27 women, participated in this study. The characteristics of the patients are summarized in Table 1.

### 3.2. Provocation of Headache by MPT

Familiar headaches were provoked in all 30 patients, but not at all sites. The number of locations provoking the familiar headaches ranged from 1 to all 8 locations (median: 5, IQR: 3–7).

Table 2 shows the percentage of provocation of headaches for all eight sites.

During this provocation, mean thumb pressures ranged from 0.81 to 1.2 kg/cm2 across all measurements.

### 3.3. Reduction of Headache by Sustained MPT

The reduction of headaches during three consecutive repetitions of MPT was measured via the headache intensity and the time needed for the reduction to occur.

#### 3.3.1. Headache Intensity

**Figure 3:**
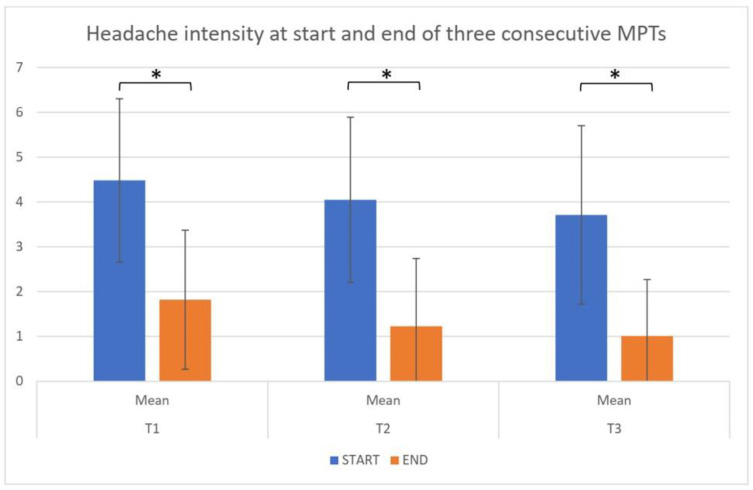
Headache intensity (numerical pain rating score, 0–10) at the start and end of each of the three consecutive trials (T1, T2, T3). MPTs = Manual Pressure Techniques * = *p* < 0.001 (paired T-test).

In 23 patients, changes in headache intensity were monitored at sites where the familiar headaches were reproduced. In 21 patients, there was a reduction in headache intensity up to an NPRS of <2/10 at the end of the last repetition. The mean headache intensity decreased during the first repetition from 4.48 points ± 1.82 points at the start to 1.82 points ± 1.55 points at the end; during the second repetition from 4.05 points ± 1.84 points at the start to 1.23 points ± 1.51 points at the end; and during the third repetition from 3.71 points ± 1.99 points at the start to 1.02 points ± 1.23 points at the end. This reduction was significant (paired T-test, for all three repetitions *p* < 0.001). At the start of each of the three consecutive repetitions, the provoked headache was always slightly less intense (ANOVA, *p* = 0.05). The pain intensity at the end of the three consecutive repetitions did not differ between the three consecutive repetitions (ANOVA, *p* = 0.278). These findings are visually represented in Figure 3.

#### 3.3.2. Time for Reduction to Occur

**Figure 4:**
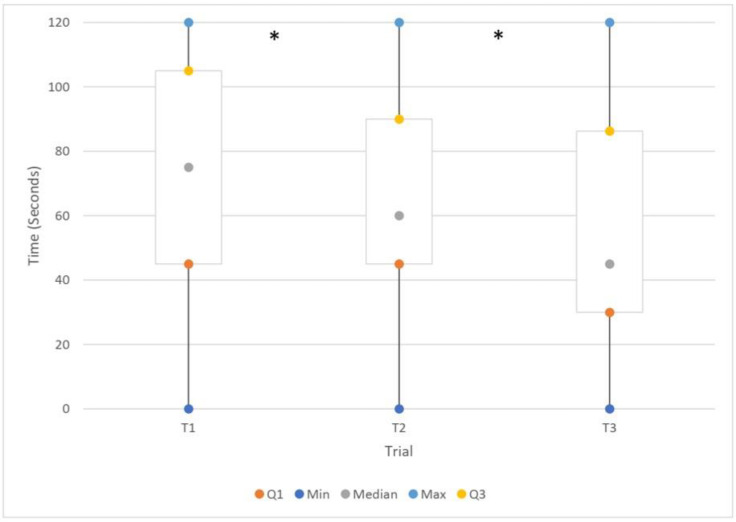
Time (seconds) needed to obtain a reduction of the referred head pain in three consecutive trials (T1, T2, T3). *: significant difference (Wilcoxon signed-rank test).

With the three repetitions, the decrease in headache intensity occurred faster. A significant difference in time (seconds) was found between the first and second repetitions (Wilcoxon signed-rank test, *p* = 0.002), the second and the third (Wilcoxon signed-rank test, *p* = 0.002), and between the first and the third (Wilcoxon signed-rank test, *p* < 0.001). This is represented visually in Figure 4.

### 3.4. Registered Pressure

The registered pressure during the sustained MPT did not differ significantly between the first, second, and third trials (see Table 3).

**Figure 5:**
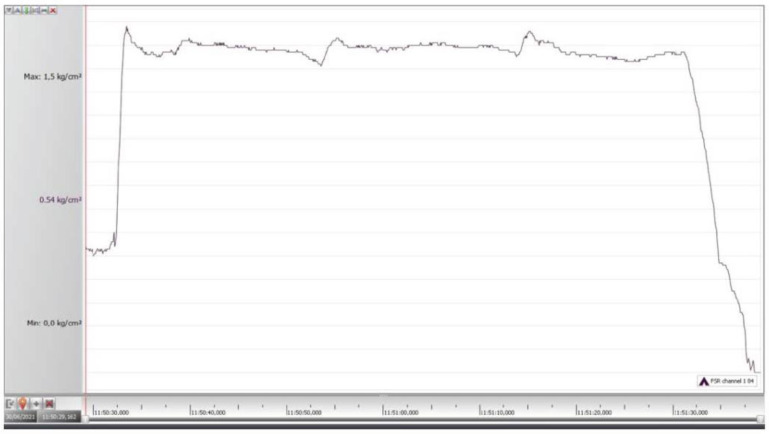
Example of thumb pressure during the manual pressure technique.

During each application, a variation (decline or increase) of 9% in thumb pressure occurred. An example of a thumb pressure/time graph is presented in Figure 5.

## 4. Discussion

This study showed that a manual pressure with a low force can provoke referred pain to the head in patients with migraine in the interictal phase. Sustaining the MPT results in a reduction in the referred head pain. Repeating the MPT results in less intense pain provocation and a faster reduction in referred head pain.

The provocation of referred head pain occurred even with low pressures. The maximum was only 1.78 kg/cm^2^. This is low compared to, e.g., the 4 kg/cm^2^ reference value in the tender point examination from The American College of Rheumatology’s diagnostic criteria for fibromyalgia [26]. The fact that such low pressures can induce referred pain to the head suggests increased mechano-sensitivity in the upper-cervical region and an altered central pain processing in the migraine [11] It is likely that the upper cervical region was already sensitive since having neck pain was an inclusion criterion.

Our MPTs are similar to those applied by Watson et al. [14,17] and Luedtke [15]. By combining pressure and movement in the upper cervical region, pressure is applied to articular as well as myofascial structures, such as the suboccipital muscles, rectus capitis posterior major, and obliquus capitis inferior muscles. Consequently, they can all serve as sources of nociceptive stimulation on the trigeminocervical system.

A recent study found that patients with migraine where head pain could be provoked on palpation were more likely to improve after a physiotherapy intervention [16]. This may indicate that modulation of nociceptive transmission at the TCC may be of potential benefit in treating the neck in patients with migraine.

Provocation of referred pain to the head was more frequent at higher cervical levels. The high percentage (93%) of provocation at C1 is in line with Watson and Drummond [14]. Comparisons with the literature for the MPTs at the rectus capitis posterior major muscles and inferior oblique muscles are hard to make since these techniques are not yet described. However, the provocation of characteristic-referred head pain by muscle tissues is described in studies investigating active trigger points in patients with migraine [27,28]. The observed provocation of referred head pain is assumed to be caused by a combination of stretch and applied pressure on myofascial tissues (muscles, ligaments), resulting in increased nociceptive input to second-order neurons at the pars caudalis of the trigeminal–cervical complex. We cannot exclude that the greater occipital nerve was stimulated during the MPT at the rectus capitis posterior major muscles. However, since the provocation rate was also high with the first MPT (pressure on the arch of C1), it appears that the higher occurrence cannot be solely related to stimulation of the greater occipital nerve. All manual pressure techniques were applied in the upper cervical area with a direct neuro-anatomical relation and nociceptive impact on the trigeminal–cervical complex [11]. 

We not only provoked the familiar head pain but reduced it by sustaining and repeating the MPTs three times. Similar to the study by Watson and Drummond, a significant reduction of the provoked pain intensity between the start and end of each trial occurred. Since all our participants had neck pain, it was not unlikely that the upper cervical region was already sensitive. Thus, even in an already sensitive starting position, the provoked referred pain can decrease. During the application of the MPTs, we monitored how many seconds it took to obtain this reduction. In the three consecutive trials, this reduction occurred faster. One could argue that during the MPT, the thumb pressure decreased, resulting in a less provocative stimulus. However, we registered a pressure variation of only 9%. Compared to the distribution of normative data from the pincer grip, this is a minimal variation [29]. Therefore we are confident that the reduction of headaches during the MPTs cannot be explained by a decrease in the applied pressure. When the procedure ended and the manual pressure was released, the provoked headache and local pain faded and resolved completely.

The characteristic headache could be provoked on both sides. This can be explained by the side-shifting character of the migraine. In our sample, about half of the patients did not have dominant headache sides. The provocation of familiar headaches on both sides may imply that hyperexcitability at the level of the spinal dorsal horn of C1-2 is not restricted to one side. The dysfunctional activity of supraspinal descending inhibitory pathways may lead to hyperexcitability at multiple levels on both sides and may explain the occurrence of headaches and altered mechanosensitivity on both sides [11,30,31,32].

During the provocation and reduction of the referred pain to the head, most patients experienced their characteristic headaches as during a migraine attack. Further, the referred pain to the head showed a pattern of referred pain that appeared to be identical to the characteristics of somatic referred pain, as explained by the hyperexcitability model described by Graven-Nielsen [31]. According to the key points of somatic referred pain, during the trials we observed that (i) a firm local and painful stimulus on myofascial structures was able to initiate, with a short time delay, a referred pain sensation in a distant somatic structure, (ii) was felt as a deep sensation, (iii) appeared to be semi-directional (i.e., from the neck to the head), and (iv) that the referred pain inhibited over time and diminished before the local pain [31]. 

This provocation and reduction of referred pain may indicate modulation of central neural transmission of nociception. Sustained MPTs are supposed to affect the nociceptive modulating system by constant and repeated pressure on specific locations in the upper cervical region, and affecting the TCC through afferent pathways. The neurophysiological and behavioral backgrounds of the inhibitory effects of repetitive pain stimuli are described in different models, such as counterirritation and habituation [33,34,35]. Activation of descending nociceptive inhibitory pathways has been determined in both concepts. The typical decrease of initially referred headache by each, and after three (our study) or four trials [17] of sustained MPT, showed similar characteristics as described for conditioned pain models, suggesting activation of descending nociceptive inhibitory pathways.

To perform all measurements interictally, measuring moments were planned at least two days after the last migraine attack. Within the provided time frame of our study, we assessed the effects of the sustained MPTs in 23 patients. We received no information that the applied techniques provoked migraine.

Our study has some limitations: All our patients suffered from migraine, diagnosed according to the ICHD III criteria for migraine by a neurologist or general practitioner, and neck pain. This reflects the characteristics of patients who asked for cervical spine treatment in daily practice [36]. However, the co-occurrence of neck pain may have led to the preselection of patients who were more sensible to the provocation of referred head pain. Compared to Watson and Drummond, in a similar percentage of patients, the characteristic headache could be provoked by the MPT [14]. In the sample from Luedtke and May, where neck pain was not an inclusion criterion, the characteristic headache was only provoked in 47% of cases [15]. Therefore, we cannot rule out that patients’ expectations and other psychological factors may have influenced the results. Although most patients with migraine experience neck pain, we cannot generalize our findings to all patients with migraine. Consequently, repetition of our study in a different setting is needed to confirm our results.

Future studies are also needed to verify if the pressure in the upper cervical spine can provoke characteristic headaches in patients with migraine without concomitant neck pain. Although modulation of nociception at the trigeminal cervical complex appears to be involved in the provocation and reduction of referred head pain by MPT, more research is needed to understand and clarify the neurophysiological background of this phenomenon. Since some preventive medications can result in chronic neuromodulation, information on the intake of preventive medication should be collected and reported in future trials.

We did not perform any visual, vestibular, or somatosensory measurements in our current study. In future studies, this could be useful to study the role of the vestibular nucleus complex in migraine symptomatology, such as in vestibular migraine [37]. For instance, Tjell et al. found a connection between chronic multi-canal benign paroxysmal positional vertigo and symptoms such as headaches [38]. Moreover, Carvalho et al. found that patients with migraine, and in particular patients with chronic migraine, had greater balance dysfunctions, even in the absence of otoneurologic abnormalities [39].

Due to the lack of numerical data, no a priori sample size calculation could be performed. Therefore, we opted for a convenience sample of 30 subjects, which is double the number of subjects in the study by Watson et al. [17]. Since we provide numerical data, our study can serve as a basis for power calculations for future studies.

We only repeated the MPTs three times in one session, and can, consequently, only mention a short-duration effect. It is not yet known if more frequent repetitions of the MPTs in multiple sessions could have longer therapeutic effects. Further research is needed to determine if MPTs can be of value in treatment programs to reduce headaches in patients with migraine. At least one trial is currently ongoing [40].

## 5. Conclusions

MPTs provoke referred pain to the head in patients with migraine. Provocation and reduction of referred pain were obtained, indicating modulation of central nociceptive transmission. The neurophysiological mechanism of provocation and the reduction of headaches by sustained MPT needs to be further elucidated.

## Figures and Tables

**Table 1:** Demographic data and headache characteristics of patients with migraine (*n* = 30) * = headache diary.

Demographics	
**Gender (male/female)**	3/27
Age in years, mean (SD)	41 (13.4)
**Headache characteristics**	
Length of history of migraine in years, mean (SD)	21.5 (14.9)
Headache pain intensity (numerical pain rating scale) (*), mean (SD)	6.2 (1.4)
Frequency in days per month (*), mean (SD)	8.9 (6.4)
Headache Impact Test 6, mean (SD)	63.4 (4.2)
**Pain measurements**	
Central sensitization index A, mean (SD)	42.9 (12.6)
Pressure pain thresholds in kPa, mean (SD)	
Midpoint upper trapezius muscle, left side	290.85 ± 168.51
Thenar, left side	295.68 ± 131.78
Anterior tibial muscle, left side	399.85 ± 289.00
Midpoint upper trapezius muscle, right side	312.19 ± 200.54
Thenar, right side	318.37 ± 125.28
Anterior tibial muscle, right side	380.70 ± 257.23

**Table 2:** Provocation of familiar headaches and corresponding mean numerical pain rating scale (NPRS) and registered pressure (kg/cm^2^). SD = standard deviation.

Measured Site		Overall % Provoked	NPRS (Mean ± SD)	Registered Pressure (kg/cm^2^, Mean ± SD)
C0-1	Left side	76.7%	5.04 ± 1.8	0.93 ± 0.50
	Right side	76.7%	5.48 ± 1.8	1.00 ± 0.35
	One of both sides	93.3%		
C2-3	Left side	43.3%	5.62 ± 1.7	0.82 ± 0.35
	Right side	53.3%	5.44 ± 1.9	0.99 ± 0.40
	One of both sides	56.7%		
Rectus Capitis Major muscle	Left side	63.3%	5.00 ± 1.7	1.17 ± 0.42
	Right side	70.0%	4.57 ± 1.8	1.13 ± 0.47
	One of both sides	**83.3%**		
Oblique Inferior Muscle	Left side	53.3%	3.4 ± 1.5	0.95 ± 0.51
	Right side	50.0%	4.87 ± 2.0	0.85 ± 0.53
	One of both sides	70.0%		

**Table 3:** Comparison of the pressure applied during the MPTs in three consecutive trials (* Friedman test).

Measured Site		Trial 1 kg/cm^2^	Trial 2 kg/cm^2^	Trial 3 kg/cm^2^	*p*-Value *
C1	Left side	0.77 ± 0.35	0.79 ± 0.38	0.81 ± 0.39	0.34
	Right side	0.99 ± 0.46	0.83 ± 0.46	0.87 ± 0.40	0.21
C2-3	Left side	0.81 ± 0.038	0.76 ± 0.32	0.82 ± 0.32	0.61
	Right side	0.79 ± 0.47	0.87 ± 0.47	0.85 ± 0.44	0.46
Rectus Capitis Posterior Major muscle	Left side	0.97 ± 0.45	0.99 ± 0.47	0.96 ± 0.46	0.76
	Right side	1.04 ± 0.50	1.09 ± 0.53	0.99 ± 0.52	0.34
Oblique Inferior Muscle	Left side	0.87 ± 0.43	0.85 ± 0.52	0.97 ± 0.46	0.61
	Right side	0.92 ± 0.43	0.91 ± 0.47	0.94 ± 0.52	0.76

## Data Availability

The datasets used and/or analyzed during the current study are available from the corresponding author upon reasonable request.
